# Low Serum Magnesium Levels Are Associated With Hemorrhagic Transformation After Thrombolysis in Acute Ischemic Stroke

**DOI:** 10.3389/fneur.2020.00962

**Published:** 2020-09-02

**Authors:** Zicheng Cheng, Xiaoyan Huang, Farah Mohamed Muse, Lingfan Xia, Zhenxiang Zhan, Xianda Lin, Yungang Cao, Zhao Han

**Affiliations:** ^1^Department of Neurology, The Second Affiliated Hospital and Yuying Children's Hospital of Wenzhou Medical University, Wenzhou, China; ^2^Department of Neurology, The Wenzhou Third Clinical Institute Affiliated to Wenzhou Medical University, Wenzhou, China

**Keywords:** magnesium, hemorrhagic transformation, thrombolysis, recombinant tissue plasminogen activator, acute ischemic stroke

## Abstract

**Background:** In patients with acute ischemic stroke, hemorrhagic transformation is a major complication after intravenous thrombolysis. This study aimed to investigate the relationship between serum magnesium levels and hemorrhagic transformation (HT) after thrombolytic therapy.

**Methods:** We retrospectively analyzed data from 242 patients who received thrombolytic therapy at the Second Affiliated Hospital of the Wenzhou Medical University in China. Baseline serum magnesium levels were measured before intravenous thrombolysis, and the occurrence of HT was evaluated using computed tomography images reviewed within 24–36 h after therapy. The relationship between serum magnesium levels and HT was examined using multivariate logistic regression, subgroup analysis, and restricted cubic spline models.

**Results:** Of the 242 included patients, 43 (17.8%) developed HT. Patients with HT had significant lower serum magnesium levels than those without HT (0.81 ± 0.08 vs. 0.85 ± 0.08 mmol/L, *p* = 0.007). Multivariable logistic regression analysis indicated that patients with higher serum magnesium levels had lower risk of HT (OR per 0.1-mmol/L increase 0.43, 95% CI 0.27–0.73, *p* = 0.002). However, this association did not persist when baseline levels of serum magnesium were higher than the median value (0.85 mmol/L) in subgroup analysis (OR per 0.1-mmol/L increase 0.58, 95% CI 0.14–2.51, *p* = 0.47). This threshold effect was also observed in the restricted cubic spline model when serum magnesium levels were above 0.88 mmol/L. No association between symptomatic HT and serum magnesium levels was observed in our study (OR per 0.1-mmol/L increase 0.52, 95% CI 0.25–1.11, *p* = 0.092).

**Conclusions:** Lower serum magnesium levels in patients with ischemic stroke are associated with an increased risk of HT after intravenous thrombolysis, but perhaps only when serum magnesium is below a certain minimal concentration.

## Introduction

Intravenous thrombolysis (IVT) with recombinant tissue plasminogen activator (rt-PA) is the preferred treatment for acute ischemic stroke patients in super earlier period (≤ 4.5 h) (1). However, hemorrhagic transformation (HT) is common in patients with IVT, occurring as a consequence of coagulation dysfunction and blood-brain barrier (BBB) disruption induced by rt-PA (2, 3). Limited administration of thrombolytic drugs in stroke patients is largely due to the fear of HT. Since patients with HT are susceptible to early death or long-term disability (4, 5), it is imperative to identify the modifiable risk factors of HT after IVT.

Magnesium is an abundant endogenous neuroprotective agent that has close association with ischemic stroke (6). It also maintains the integrity of the vascular endothelial barrier through anti-inflammatory and anti-oxidation effects (7). Brain microvascular endothelium is the fundamental component of BBB and the initiation of ischemia-related BBB disruption is predominantly triggered by endothelial damage (8). Recent evidence indicates the protective effect of magnesium on BBB in rats with transient focal cerebral ischemia (9). In addition, magnesium is involved in the coagulation cascade (10, 11) and platelet activation (12); magnesium deficiency would lead to dysfunction of coagulation system. The relationship between serum magnesium levels and functional outcomes in patients with acute ischemic stroke has been widely studied. But two large-sample randomized controlled trials [the IMAGES (Intravenous Magnesium Efficacy in Stroke) trial and the FAST-MAG (Field Administration of Stroke Therapy–Magnesium) trial] showed regrettable results, which early intravenous magnesium sulfate therapy did not improve the outcomes for patients with acute stroke (13, 14). By contrast, there are very few studies on the association between serum magnesium levels and the occurrence of HT. These studies had inconsistent conclusions and did not specifically address patients with IVT (15, 16). Thus, we investigated the association of serum magnesium levels with development of HT in patients with acute ischemic stroke after IVT.

## Methods

### Patients

This retrospective, observational, single-center study was conducted at the Second Affiliated Hospital of the Wenzhou Medical University, China. All ischemic stroke patients who received IVT at the Department of Neurology between January 2015 and January 2020 were evaluated for eligibility. All the included patients received a confirmed diagnosis of acute ischemic stroke via magnetic resonance imaging (MRI) or computed tomography (CT), and received rt-PA infusion of 0.9 mg/kg (a maximum of 90 mg) on arrival in the emergency room. We excluded patients with no available data on baseline serum magnesium levels or no follow-up CT images within 24–36 h after IVT. This study was approved by the Ethics Committee of the Second Affiliated Hospital and Yuying Children's Hospital of the Wenzhou Medical University. Written informed consent was exempted for the retrospective nature of the study.

### Data Collection

From the medical records, we obtained demographic data (age and sex), baseline clinical parameters [systolic and diastolic blood pressure, blood glucose level, international normalized ratio (INR), activated partial thromboplastin time (APTT), platelet count, serum magnesium, and serum calcium levels, National Institutes of Health Stroke Scale (NIHSS) score, onset-to-treatment time, and current antithrombotic therapy], data on vascular risk factors (hypertension, diabetes, hyperlipidemia, atrial fibrillation, coronary heart disease, history of stroke, current smoking, and current drinking), and acceptance of bridging therapy, as well as additional laboratory data [total cholesterol, low-density lipoprotein cholesterol (LDL-C), and HbA1c]. All patients underwent CT scanning during admission, as well as within 24–36 h after IVT. Antithrombotic agents were administered to all patients only after a follow-up CT was conducted.

### Assessment of Hemorrhagic Transformation

All CT images were retrospectively evaluated by two experienced neurologists who were blinded to patients' clinical data, and disagreements were settled by further discussion. Based on the European Cooperative Acute Stroke Study (ECASS) criteria, we classified HT into hemorrhagic infarctions (HI 1 or 2) and parenchymal hemorrhage (PH 1 or 2) (17). Symptomatic hemorrhagic transformation (sHT) was defined as HT accompanied by neurological deterioration (18).

### Statistical Analysis

Continuous variables are presented as mean and standard deviations in the case of normally distributed data, or medians (interquartile range) in the case of skewed data. Differences in continuous variables were analyzed using unpaired *t*-tests or Mann–Whitney *U*-tests. Categorical variables are presented as percentages. Inter-group differences were analyzed using Pearson's chi-square test or Fisher's exact probabilities test. Measured serum magnesium levels were collapsed into quartiles, and the first quartile values were used as the reference category for the logistic regression analysis. To examine a potential independent association between serum magnesium levels and development of HT, two multivariate logistic regression models were evaluated: model 1 adjusted for all variables with *p* < 0.1 in the univariate analysis, while model 2 tested the variables from model 1 along with other potential risk factors of HT (age, hypertension, and baseline blood glucose levels), identified based on the literature (19). Serum magnesium concentrations were entered into the models in two formats, as a continuous variable (in which case OR was calculated per 0.1 mmol/L increase) or as a four-categorized variable (in which case OR was calculated compared to the first quartiles).

A subgroup analysis was conducted after stratifying the patients using the median value of baseline serum magnesium levels as a cut-off. In addition, we used the R package “rms” to construct restricted cubic splines with three knots to understand patterns in the association between serum magnesium levels and HT, using the median value of baseline serum magnesium as a reference point (20). All statistical tests were two-tailed, and *p* < 0.05 was considered statistically significant. All analyses were performed using SPSS 25.0 (IBM, Armonk, NY, USA) and R 3.6.3 (R Foundation for Statistical Computing, Vienna, Austria).

## Results

A total of 268 acute ischemic stroke patients who received IVT treatment were screened for eligibility. After excluding patients with no available data on baseline serum magnesium levels (*n* = 21) or follow-up CTs (*n* = 5), we analyzed clinic data from 242 patients to determine the effect of serum magnesium levels on the development of HT. Mean patient age was 68.6 ± 14.1 years, and 154 (63.6%) were men. Among the included patients, 43 (17.8%) presented with HT, comprising 15 (6.2%) with HI 1, 13 (5.4%) with HI 2, 10 (4.1%) with PH 1, and 5 (2.1%) with PH 2. Of these 43 patients, 13 (5.4%) developed sHT.

The average concentration of serum magnesium across all patients was 0.84 ± 0.08 mmol/L. Serum magnesium levels in patients who developed HT were significantly lower than levels in those who did not develop HT (0.81 ± 0.08 vs. 0.85 ± 0.08 mmol/L, *p* = 0.007, Table 1). Patients with HT also had a significantly higher incidence of atrial fibrillation and bridging therapy, higher INRs and NIHSS scores, and lower LDL-C levels than those without HT (Table 1). Multivariate logistic regression showed that, after adjusting for potential confounding factors, serum magnesium levels had an independent negative relationship with HT risk (OR per 0.1-mmol/L increase 0.43, 95% CI 0.27–0.73, *p* = 0.002; Table 2). Patients in the third and fourth serum magnesium quartiles showed a significant decline in HT risk (OR 0.18, 95% CI 0.05–0.60, *p* = 0.005; OR 0.33, 95% CI 0.12–0.92, *p* = 0.035; respectively) compared to those in the first quartile.

**Table 1:** Clinical characteristics of thrombolytic patients, stratified by the development of HT.

	**No HT (*n* = 199)**	**HT (*n* = 43)**	***P***
Age in years, mean ± SD	68.1 ± 14.2	70.8 ± 13.6	0.25
Female, *n* (%)	71 (35.7%)	17 (39.5%)	0.63
Hypertension, *n* (%)	151 (75.9%)	34 (79.1%)	0.66
Diabetes mellitus, *n* (%)	59 (29.6%)	13 (30.2%)	0.94
Hyperlipidemia, *n* (%)	81 (40.7%)	14 (32.6%)	0.32
Atrial fibrillation, *n* (%)	53 (26.6%)	27 (62.8%)	** <0.001**
Coronary artery disease, *n* (%)	18 (9.0%)	6 (14.0%)	0.33
History of stroke, *n* (%)	24 (12.1%)	7 (16.3%)	0.45
Current Smoking, *n* (%)	47 (23.6%)	11 (25.6%)	0.78
Current Drinking, *n* (%)	36 (18.1%)	9 (20.9%)	0.66
Current antithrombotic therapy, *n* (%)	28 (14.1%)	11 (25.6%)	0.063
OTT in min, median (IQR)	169 (125–210)	180 (125–205)	0.84
Baseline NIHSS score, median (IQR)	8 (4–13)	13 (8–19)	** <0.001**
Baseline SBP in mm Hg, mean ± SD	156.6 ± 21.2	156.9 ± 23.1	0.93
Baseline DBP in mm Hg, mean ± SD	87.2 ± 15.3	89.5 ± 16.9	0.37
Baseline blood glucose in mmol/L, median (IQR)	6.91 (5.95–8.98)	7.27 (6.47–9.69)	0.10
Platelet count in 10^9^/L, median (IQR)	191 (167–238)	190 (154–219)	0.24
INR, median (IQR)	1.03 (0.99–1.09)	1.07 (1.04–1.14)	** <0.001**
APTT in sec, median (IQR)	33.8 (30.6–36.6)	33.3 (31.7–36.0)	0.94
Serum magnesium in mmol/L, mean ± SD	0.85 ± 0.08	0.81 ± 0.08	**0.007**
Serum calcium in mmol/L, mean ± SD	2.25 ± 0.11	2.22 ± 0.11	0.081
HbA1c in %, median (IQR)	5.90 (5.50–6.50)	6.07 (5.70–6.70)	0.40
TC in mmol/L, median (IQR)	4.40 (3.79–5.10)	4.11 (3.53–4.76)	0.12
LDL_C in mmol/L, median (IQR)	2.60 (2.07–3.32)	2.35 (1.84–2.75)	**0.033**
Bridge therapy, *n* (%)	17 (8.5%)	8 (18.6%)	**0.049**

*APTT, activated partial thromboplastin time; DBP, diastolic blood pressure; HT, hemorrhagic transformation; INR, international normalized ratio; IQR, interquartile range; LDL-C, low-density lipoprotein cholesterol; NIHSS, National Institutes of Health Stroke Scale; OTT, onset-to-treatment time; SBP, systolic blood pressure; SD, standard deviation; TC, total cholesterol. The bold values represent significant results in univariate analysis (P < 0.05)*.

**Table 2:** Multiple logistic regression analysis to identify relationships between serum magnesium levels and risk of hemorrhagic transformation after thrombolysis.

	**Model 1**		**Model 2**	
	**OR (95% CI)**	***P***	**OR (95% CI)**	***P***
Serum magnesium, per 0.1-mmol/L increase	0.44 (0.27–0.73)	0.001	0.43 (0.27–0.73)	0.002
Serum magnesium, quartiles				
Q1 (<0.80 mmol/L)	Reference		Reference	
Q2 (0.80–0.84 mmol/L)	0.40 (0.15–1.04)	0.060	0.45 (0.17–1.23)	0.12
Q3 (0.85–0.90 mmol/L)	0.32 (0.12–0.88)	0.029	0.33 (0.12–0.92)	0.035
Q4 (> 0.90 mmol/L)	0.18 (0.06–0.58)	0.004	0.18 (0.05–0.60)	0.005
*P* for trend	0.002		0.003	

*Model 1: adjusted for atrial fibrillation, ongoing antithrombotic therapy, baseline NIHSS score, INR, serum calcium (per 0.1-mmol/L increase), LDL-C, and bridging therapy*.

*Model 2: adjusted for the variables in model 1, as well as age, hypertension, and baseline blood glucose levels*.

*CI, Confidence interval; INR, international normalized ratio; LDL-C, low-density lipoprotein cholesterol; NIHSS, National Institutes of Health Stroke Scale; OR, Odds ratio*.

**Figure 1:**
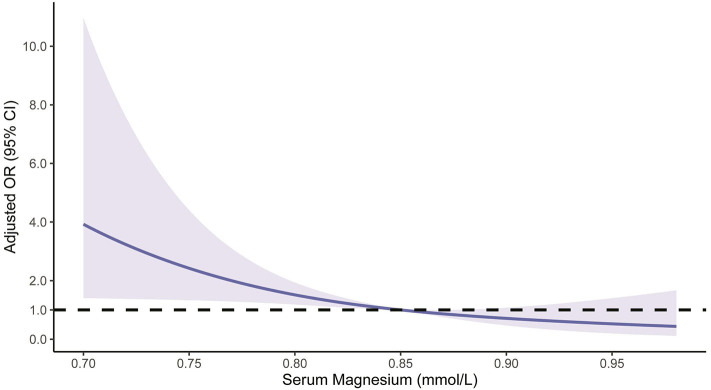
Restricted cubic spline regression model of the relationship between serum magnesium levels and risk of hemorrhagic transformation. The solid blue line represents the odds ratio (OR), and the blue shading depicts the corresponding 95% confidence interval (CI). The dotted black line is the reference line for which OR = 1. The model was adjusted for the same confounding variables as in model 1 in Table 2.

Patients were then stratified based on the median serum magnesium level of 0.85 mmol/L. Subgroup analysis showed a negative association between serum magnesium levels and HT risk when the levels were below the median value (OR per 0.1-mmol/L increase 0.33, 95% CI 0.12–0.93, *p* = 0.037), but no association when serum magnesium levels were above the median value (OR per 0.1-mmol/L increase 0.58, 95% CI 0.14–2.51, *p* = 0.47; Table 3). The restricted cubic spline model corroborated the significant negative linear relationship between serum magnesium and HT, but also showed that ample serum magnesium levels did not further lead to a reduction in HT risk [the reference line (OR = 1) cross the corresponding 95% CI of OR when serum magnesium levels were higher than 0.88 mmol/L, Figure 1].

**Table 3:** Stratified logistic regression analysis to identify relationships between serum magnesium levels and risk of hemorrhagic transformation after thrombolysis.

	**OR (95% CI)**	***P***
Serum magnesium, per 0.1-mmol/L increase		
< median (<0.85 mmol/L)	0.33 (0.12–0.93)	0.037
≥median (≥ 0.85 mmol/L)	0.58 (0.14–2.51)	0.47

*The model was adjusted for the same confounding factors as in model 1 in Table 2*.

*CI, Confidence interval; OR, Odds ratio*.

Based on the univariate analysis, we found that patients with sHT had insignificantly lower serum magnesium levels compared to those who did not develop sHT (0.81 ± 0.10 vs. 0.84 ± 0.08 mmol/L, *p* = 0.21; Table S1). After adjusting for confounding factors, our analysis showed that high serum magnesium levels did not lead to a significant decrease in sHT risk (OR per 0.1-mmol/L increase 0.52, 95% CI 0.25–1.11, *p* = 0.092; Table S2). Serum magnesium levels were similar between HI and PH (0.81 ± 0.08 vs. 0.81 ± 0.09 mmol/L, *p* = 0.87).

## Discussion

This retrospective study evaluated the association between serum magnesium levels and occurrence of HT in 242 acute ischemic stroke patients after IVT treatment. Our findings indicate that there is a significant association between low serum magnesium levels at admission and an increased risk of developing HT within 24–36 h after IVT. However, this negative relationship does not appear to hold when serum magnesium levels are above a certain minimum.

How serum magnesium concentrations influence the risk of HT in stroke patients after IVT remains unclear. The reason may relate to the role of magnesium in neuroprotection and hemostasis. Rt-PA can cause post-stroke HT by inducing oxidative stress that further compromise the BBB (3), and magnesium-deficiency would lead to enhanced permeability of the cerebrovascular endothelial barrier as proved in mouse models (7). Intraperitoneal injections with magnesium sulfate in normal mice reduced histamine-induced endothelial hyper-permeability mainly by suppressing oxidative stress and inflammatory responses, while up-regulating endothelial barrier-stabilizing mediators such as cyclic adenosine monophosphate (7). In rats subjected to transient focal cerebral ischemia, magnesium showed the ability to protect BBB integrity by increasing the activity of anti-oxidant enzymes and decreasing lipid peroxide levels (9). Another pivotal pathogenesis of HT after IVT is rt-PA induced coagulopathy (2, 21). Magnesium can shorten prothrombin time by accelerating the activation of factor X through the factor IXa- and factor VIIa-tissue factor-mediated pathways (10, 22). Magnesium also promotes the adherence of platelets to collagen, independent of platelet activation, and secretion (12). In patients with intracerebral or subarachnoid hemorrhage, magnesium shows hemostatic properties: serum magnesium levels are negatively associated with hematoma volume (23–25).

Similarly to our study, an increase in risk of HT in patients with low serum magnesium levels was observed in another Chinese patient sample (16). Differently, thrombolytic patients were not included, besides association between adequate serum magnesium levels and HT was not analyzed in their study (16). Our finding that the association between serum magnesium levels and the occurrence of HT after thrombolysis disappeared at serum magnesium concentrations ≥0.88 mmol/L may reflect a threshold effect for the capacity of magnesium to prevent HT. In fact, Dong et al. (26) found that magnesium, at physiological levels, could maintain integrity for human endothelial cells *in vitro*, while extra magnesium did not bring about an increase in integrity. This suggests that the maximal protective effect of magnesium on the BBB may appear within the physiological concentration range. Moreover, although magnesium is an essential component to the coagulation system, *in vitro* studies suggest that excessive magnesium levels may inversely prolong prothrombin time (10) and inhibit human platelet function (26, 27). Decreased platelet activity and increased bleeding time have also been observed in healthy volunteers given intravenous magnesium sulfate, accompanied by an elevation in mean serum magnesium concentrations from 0.85 to 1.50 mmol/L (28). These findings are consistent with our results, suggesting that the risk of HT does not decline further after serum magnesium levels exceed a certain value.

Our findings imply that magnesium supplementation may reduce risk of HT in patients undergoing IVT, at least up to a certain point. By contrast, the IMAGES and FAST-MAG clinical trials showed that magnesium sulfate treatment did not improve outcomes for stroke patients (13, 14). Researchers attributed the failure to the delay of magnesium in crossing the BBB, so that the concentration did not accumulate immediately in brain tissues (14). It is noteworthy that the patients included in both their studies were not only ischemic stroke, but hemorrhagic stroke and stroke mimics, besides only part of ischemic stroke patients received thrombolytic therapy. Also, Patients were randomized to receive intravenous magnesium sulfate or placebo regardless of the baseline serum magnesium levels. Therefore, it is still necessary to explore whether magnesium supplementation therapy can improve the prognosis of thrombolytic patients with low serum magnesium levels, which is mediated by the protective effect on preventing HT.

In our study, no statistically significant reduction of serum magnesium levels was observed in patients with sHT. This is different from the positive findings that serum magnesium levels affect the risk of HT after thrombolysis. A reasonable explanation is that low serum magnesium levels are associated with larger infarct volume (9, 29). Thus, subsequent hemorrhage in ischemic lesions are not sufficient to cause further deterioration of neurological function. Nonetheless, the negative result in our study may also be a type II error caused by our small sample (30). Among FAST-MAG patients, only a portion of whom was given thrombolysis, sHT was less frequent in those treated with magnesium sulfate than those who receive placebo (2.1 vs. 3.3%, *p* = 0.12) (14). Although *post-hoc* analysis of the FAST-MAG trial concluded that serum magnesium levels measured within 24 h after IVT were not associated with sHT (15), it should be noted that the sHT and non-sHT patients in their study showed higher average serum magnesium levels than physiological concentrations, and the neutral findings may result from that the additional magnesium did not further affect risk of sHT because of the threshold effect mentioned above. Given adverse outcomes of sHT are quite serious, further studies are needed to verify whether a relationship exists between serum magnesium and risk of sHT.

There are some limitations to this study. Our results should be interpreted with caution since we measured total magnesium rather than ion magnesium, which plays direct physiological roles. The single-center, retrospective design, and limited sample size restricted our ability to identify the effects of serum magnesium levels on patients affected by HT, especially sHT. The relationship between serum magnesium and sHT remains unclear. Additionally, we could not evaluate and correct for potentially confounding neuroimaging metrics such as ischemic core volume before thrombolysis and cerebral microbleeds. Regrettably, due to the observational nature of this study, serum magnesium concentrations beyond the upper normal limit were not obtained and the efficacy and safety of excess magnesium supplementation for thrombolytic patients could not be fully evaluated.

Despite these limitations, our results suggest that lower baseline serum magnesium levels are associated with increased risk of HT in acute ischemic stroke patients after IVT, but this association does not appear to hold when serum magnesium reaches a certain physiological concentration. Further randomized controlled trials are needed to determine whether early initiation of magnesium supplementation therapy in thrombolytic patients with low serum magnesium levels can reduce their risk of HT and, subsequently, improve their prognosis.

## Data Availability Statement

The raw data supporting the conclusions of this article will be made available by the authors, without undue reservation.

## Ethics Statement

The studies involving human participants were reviewed and approved by The Ethics Committee of the Second Affiliated Hospital and Yuying Children's Hospital of the Wenzhou Medical University. Written informed consent for participation was not required for this study in accordance with the national legislation and the institutional requirements.

## Author Contributions

ZH and ZC conceived and designed the study. All authors acquired the data, which ZC and FM analyzed. ZC, ZH, and XH assisted in data interpretation and wrote the manuscript. All authors participated in revising the article and approved the final version.

## Conflict of Interest

The authors declare that the research was conducted in the absence of any commercial or financial relationships that could be construed as a potential conflict of interest.
