# Supplementary material for: Low Serum Magnesium Levels Are Associated With Hemorrhagic Transformation After Thrombolysis in Acute Ischemic Stroke
Source: Front Neurol. 2020 Sep 2;11:962. doi: 10.3389/fneur.2020.00962 (PMC7492199; doi:10.3389/fneur.2020.00962)
Supplement: Supplementary file 1 [file Data_Sheet_1.docx]

**Table S1.** Clinical characteristics of thrombolytic patients, stratified by the development of sHT

|  | **No sHT (n=229)** | **sHT (n=13)** | ***P*** |
| --- | --- | --- | --- |
| **Age in years, mean ± SD** | 68.4±14.1 | 71.1±13.4 | 0.51 |
| **Female, n (%)** | 81 (35.4%) | 7 (53.8%) | 0.24 |
| **Hypertension, n (%)** | 177 (77.3%) | 8 (61.5%) | 0.19 |
| **Diabetes mellitus, n (%)** | 69 (30.1%) | 3 (23.1%) | 0.76 |
| **Hyperlipidemia, n (%)** | 90 (39.3%) | 5 (38.5%) | 1.00 |
| **Atrial fibrillation, n (%)** | 69 (30.1%) | 11 (84.6%) | **<0.001** |
| **Coronary artery disease, n (%)**  **History of stroke, n (%)**  **Current Smoking, n (%)**  **Current Drinking, n (%)**  **Current antithrombotic therapy, n (%)**  **OTT, min, median (IQR)**  **Baseline NIHSS score, median (IQR)**  **Baseline SBP in mm Hg, mean ± SD**  **Baseline DBP in mm Hg, mean ± SD**  **Baseline blood glucose in mmol/L, median (IQR)**  **Platelets in 10^9^/L, median (IQR)**  **INR, median (IQR)**  **APTT in sec, median (IQR)**  **Serum magnesium in mmol/L, mean ± SD**  **Serum calcium in mmol/L, mean ± SD**  **HbA1c in %, median (IQR)**  **TC in mmol/L, median (IQR)**  **LDL_C in mmol/L, median (IQR)**  **Bridge therapy, n (%)** | 24 (10.5%)  28 (12.2%)  56 (24.5%)  44 (19.2%)  36 (15.7%)  170 (125-208)  8 (5-14)  156.7±21.7  87.4±15.9  7.00 (6.01-9.14)  191 (165-236)  1.04 (.99-1.10)  33.8 (30.7-36.6)  0.84±0.08  2.25±0.11  5.90 (5.50-6.54)  4.34 (3.72-5.16)  2.56 (2.00-3.28)  23 (10.0%) | 0 (0%)  3 (23.1%)  2 (15.4%)  1 (7.7%)  3 (23.1%)  160 (120-213)  12 (8-18)  156.3±18.2  91.5±8.1  7.03 (6.73-9.52)  190 (135-231)  1.10 (1.04-1.17)  33.3 (30.7-35.0)  0.81±0.10  2.22±0.14  6.10 (5.75-6.61)  4.01 (3.22-5.12)  2.50 (1.69-2.91)  2 (15.4%) | 0.37  0.22  0.74  0.47  0.45  0.72  **0.034**  0.95  0.11  0.58  0.35  **0.016**  0.63  0.21  0.33  0.44  0.24  0.35  0.63 |

APTT: activated partial thromboplastin time; DBP: diastolic blood pressure; HT: hemorrhagic transformation; INR: international normalized ratio; IQR: interquartile range; LDL-C: low-density lipoprotein cholesterol; NIHSS: National Institutes of Health Stroke Scale; OTT: onset-to-treatment time; SBP: systolic blood pressure; SD: standard deviation; TC: total cholesterol.

**Table S2.** Multiple logistic regression analysis to identify predictors for symptomatic hemorrhagic transformation

|  | **OR (95% CI)** | ***P*** |
| --- | --- | --- |
| **Atrial fibrillation**  **Baseline NIHSS score**  **INR, per 0.1 increase**  **Magnesium, per 0.1-mmol/L increase** | 11.59 (2.40-56.12)  1.03 (0.97-1.11)  1.23 (0.78-1.93)  0.52 (0.25-1.11) | 0.002  0.326  0.370  0.092 |

CI: Confidence interval; INR, international normalized ratio; NIHSS, National Institutes of Health Stroke Scale; OR: Odds ratio.
